# Experimental Study on the Effects of Cockpit Noise on Physiological Indicators of Pilots

**DOI:** 10.3390/s25134175

**Published:** 2025-07-04

**Authors:** Haiming Shen, Meiqing Hao, Jiawei Ren, Kun Chen, Yang Gao

**Affiliations:** 1 Flight Academy, Civil Aviation University of China, Tianjin 300300, China; 2 College of Safety Science and Engineering, Civil Aviation University of China, Tianjin 300300, China; 15690867290@163.com (M.H.); 19851623325@163.com (J.R.); 3 Institute of Science and Technology Innovation, Civil Aviation University of China, Tianjin 300300, China; kchen@cauc.edu.cn; 4 Sino-European Institute of Aviation Engineering, Civil Aviation University of China, Tianjin 300300, China; snower523@163.com

**Keywords:** aviation safety, cockpit, cockpit noise, sound pressure level, physiological indicators

## Abstract

Cockpit noise, as a critical environmental factor affecting flight safety, may impair pilots’ cognitive functions, leading to a decreased operational performance and decision-making errors, thereby posing potential threats to aviation safety. In order to reveal the relationship between the cockpit noise sound pressure level and pilot physiological indicators, and provide a scientific basis for cockpit noise airworthiness standards, this experiment takes pilot trainees as the research subject. Based on the principle of multimodal data synchronization, a sound field reconstruction system is used to reconstruct the cockpit sound field. Electroencephalogram (EEG), electrocardiogram (ECG), and electrodermal activity (EDA) measurements are carried out in different sound pressure level noise operating environments. The results show that with the increase in the sound pressure level, the significant suppression of α-wave activity in the occipital and parietal regions suggests that the cortical resting state is lifted and visual attention is enhanced; the enhancement of the β-wave in the frontal regions reflects the enhancement of alertness and prefrontal executive control, and the suppression of θ-wave activity in the frontal and temporal regions may indicate that cognitive tuning is suppressed, which reflects the brain’s rapid adaptive response to external noise stimuli in a high-noise environment; noise exposure triggers sustained sympathetic nerve hyperactivity, which is manifested by a significant acceleration of the heart rate and a significant increase in the mean value of skin conductance when the noise sound pressure level exceeds 70 dB(A). The correlation analysis between physiological indicators shows that cockpit noise has a multi-system synergistic effect on human physiological indicators. The experimental results indicate that noise has a significant impact on EEG, ECG, and EDA indicators.

## 1. Introduction

In the aviation field, cockpit noise, as a typical scenario of occupational noise exposure, is an important factor affecting aviation safety. Cockpit noise mainly comes from the engine, aerodynamic noise, and mechanical noise inside the aircraft [1]. Pilots are often exposed to continuous noise environments during flight missions. Long-term exposure to such high-noise environments may have a significant impact on the physiological and psychological reactions of pilots. In addition, the cockpit is a closed environment, and the particularity of the cockpit environment may aggravate the physiological interference effect of noise. The research shows that the exposure of the aircraft cabin in the take-off and landing stage is more than 70 dB(A), some more than 80 dB(A), and the cruise stage is still above 70 dB(A) [2]. Noise poses a potential threat to pilots’ hearing, and long-term exposure can lead to noise-induced hearing loss [3]. In addition, noise can cause a physiological stress response, increase heart rate and blood pressure, and increase the risk of cardiovascular disease [4,5]. In addition, cockpit noise may affect the cognitive function and attention level of pilots and reduce their response speed and judgment, which is very important for the execution of flight missions [6,7]. Long-term noise exposure may also lead to mental health problems such as fatigue, anxiety, and stress in pilots, which further affect their work performance. However, how such intermittent high-intensity noise affects pilots’ autonomic nervous regulation and cardiovascular function has not been fully elucidated.

Cockpit noise has many negative effects on human beings, but there is no relevant standard applicable to the airworthiness of a civil aircraft cockpit to clearly limit the cockpit noise, so as to reduce the physiological harm of noise to human beings. Currently, there is no uniform, specific, and quantifiable international standard for the aircraft cockpit noise environment. Although the Civil Aviation Administration of China (CAAC), the Federal Aviation Administration (FAA), and the European Aviation Safety Agency (EASA) have relevant provisions in Article 25.771 (e), which clearly states “The vibration and noise characteristics of cockpit equipment shall not affect the safe operation of the aircraft,” the provision is still a principle requirement. However, this provision is still a requirement in principle and lacks specific sound pressure level limits or frequency characteristics indicators. In terms of domestic standards, the Limits of Occupational Exposure to Hazardous Factors in the Workplace Part 2: Physical Factors (GBZ 2.2-2007) and Noise Levels in Aircraft (GJB 1357-1992) stipulate that when the noise level in the cockpit of an aircraft is more than 85 dB(A), an effective hearing protection device should be equipped to ensure that the noise attenuation is less than 85 dB(A) and that hearing risks can be controlled during the limited exposure time, so as to control the risk of hearing loss, so as to protect the pilot’s health and operational efficiency [8,9]. The influence mechanism of noise on physiological indicators is mainly achieved by interfering with the auditory system, central nervous system, and autonomic nervous system [9,10,11,12]. Although cockpit noise studies have traditionally focused on the auditory pathway, recent studies have pointed out that noise may also affect physiological responses through non-auditory mechanisms, such as vibration noise [13]. Given that headphones were used to reconstruct the sound field in this study, noise exposure was primarily limited to the auditory pathway. Noise exposure is airborne to the human ear, which subsequently triggers a series of physiological stress responses in the body that can have an impact on human physiological indicators. Due to the objectivity and measurability of physiological indicators, research methods based on physiological indicators are becoming more and more popular [14,15]. This study aims to quantify the impact of varying noise levels on key physiological indicators through signal filtering, data cleaning, and visual statistical analysis in order to reveal potential dose–response relationships between noise exposure and physiological responses.

Compared with the conventional noise exposure situation, pilots need to deal with the superposition of high-intensity noise environments and high-cognitive-load flight operation tasks in the cockpit. The dual particularity of such working environments and occupational characteristics makes the research on the physiological impact of noise on the pilot group significantly different from the conventional situation. Traditional noise impact studies focus on a single physiological dimension (as heart rate fluctuations or EEG activity characteristics) and the experimental design for pilots requires the establishment of a multi-channel synchronous monitoring system of EEG, ECG, and EDA to more fully reflect the physiological state of pilots. Through the integrated analysis of cross-modal physiological data, the synergistic analysis of neurocognition and autonomic nerve activation can be realized, so as to systematically reveal the comprehensive mechanism of noise on the pilot’s physical and mental state. The purpose of this paper is to quantify the impact of noise on physiological indicators, and to provide a scientific basis for airworthiness standards by revealing the dose–effect relationship between noise exposure and key physiological indicators. Therefore, based on the principle of multi-modal data synchronization, this paper uses the sound field restoration system to reconstruct the cockpit sound field to study the influence of different noise intensities on the multi-modal data of pilot trainees and provides a theoretical basis for the formulation of cockpit noise standards.

## 2. Materials and Methods

### 2.1. Experimental Condition

The experimental subjects were 16 pilot trainees from the Flight Academy of Civil Aviation University of China, aged 18–24 years old, all of whom were male, in good health, and had no bad habits. The subjects were asked to sleep enough in the early stage of the experiment to maintain a good mental and physical state. It has been shown that experienced flight instructors demonstrate more efficient attention allocation strategies and greater situational awareness during flight tasks, and show greater cognitive control and anti-interference capabilities when faced with complex or disruptive tasks [16]. The pilot trainees were selected as the subjects, mainly based on the feasibility of sample acquisition and experimental controllability: the student group was large and in the centralized training stage, which is convenient to control the variables; in terms of ethics, the occupational risk of participants participating in the experiment was low and their physiological systems without long-term noise exposure (such as nerve and hearing sensitivity) can more sensitively reflect the short-term effects and avoid the adaptive bias of active pilots. This study was formally reviewed and endorsed by the CAUC Flight Division Ethics Committee on 1 September 2024 (Approval No. CAUC-PSY-2024-003), which confirmed that the rights and well-being of the subjects were fully guaranteed throughout this study and that this study posed no inherent risk to the subjects.

**Figure 1:**
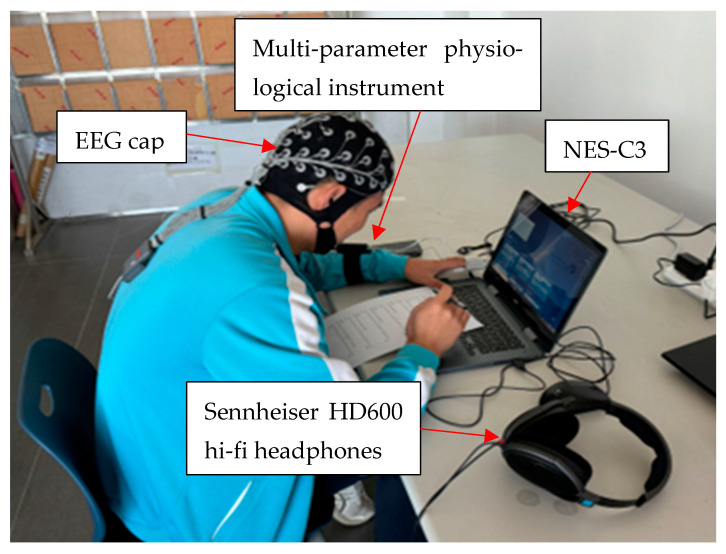
The spatial arrangement of the sound sources, measurement devices, and participant positioning.

The experiment used Siemens software (Testlab 2306 (64-Bit)), RME sound card (The manufacturer of RME sound card equipment is RME, and the equipment is sourced from Haimhausen, Germany), power amplifier (The manufacturer of power amplifier is Behringer Holdings (Pte) Ltd., and the equipment is sourced from Kirchardt, Germany), Sennheiser HD600 headphones (The manufacturer of Sennheiser headphones is Sennheiser, and the equipment is sourced from Hanover, Germany), and real flight data to form a sound field reduction system to reconstruct the sound field to simulate the environmental noise of the aircraft cockpit. The noise sources used in this study are based on real data collected during actual flights. The spatial arrangement of the sound sources, measurement equipment, and participant positioning is illustrated in Figure 1. It should be noted that the use of Sennheiser HD600 hi-fi headphones as a listening device does not provide hearing protection and no active or passive noise reduction devices were introduced during the experiment. Therefore, the experimental participants were in a state of unprotected (bare) hearing during the noise exposure and their physiological responses can be regarded as direct responses to different sound pressure levels. According to MIL-STD1474E’ Noise Limits for Military Equipment’ and the relevant noise limit standards at home and abroad, the noise level of the experiment is divided into 70 dB(A), 80 dB(A), 90 dB(A), and 40 dB(A), where 40 dB(A) is the environmental noise value of the sound field without external noise [17]. Since this experimental study is the effect of noise on the physiological indicators of the personnel, other environmental variables of the experiment are controlled separately, including temperature 26 ± 0.5 °C, relative humidity 40 ± 5% RH, and wind speed 0.2 ± 0.1 m/s, to ensure the accuracy of the experiment.

### 2.2. Physiological Data Acquisition

In this experiment, the Neuro HUB wearable multi-modal research platform was used to synchronously collect and analyze the physiological data of the subjects. The multi-modal data of the subjects could be collected synchronously under different working conditions. The EEG, ECG, and EDA data were collected synchronously by 64-channel EEG cap (sampled at a rate of 1000 Hz) and multi-parameter physiological instrument. The manufacturer of EEG map and multi-parameter physiological instrument is Neuracle, and the equipment is sourced from Changzhou, China. The EEG signal was collected by the electrode to sense the electrical activity of the brain neurons on the scalp. These activities were manifested as brain waves in different frequency bands (such as α wave, β wave, θ wave, and δ wave, etc.). As a direct characterization of central nervous system activity, the rhythmic oscillation patterns of EEG signals are highly correlated with cognitive states. It has been shown that a decrease in α-wave power reflects the weakening of neural synchronization due to enhanced external stimuli, suggesting that noisy environments force the brain to continuously process irrelevant auditory information and weaken focus on core tasks [18]; an increase in β-rhythms is usually associated with the mobilization of cognitive resources and adaptive adjustments to the difficulty of the task [19]; and θ-rhythms are usually associated with relaxation states, endogenous attention, and memory consolidation [20]. In order to deeply analyze the multidimensional regulatory characteristics of the brain’s functional state, the present study systematically integrated the EEG frequency domain ratio analysis indicators, including: θ/β reflects the level of alertness, with a decrease in the ratio indicating that the organism is in a highly alert state [21]; α/β assesses the efficiency of attention allocation, with a decreasing ratio reflecting enhanced active control and weakened spontaneous attention [22]; and β/(θ + α) characterizes the intensity of cognitive load, with elevated ratios suggesting increased neural resource depletion [23]. These empirically validated neural oscillatory coupling metrics can sensitively capture the continuous physiological spectrum of changes from basal physiological arousal to higher-order cognitive load. The heart rate and heart rate variability in different states are recorded by ECG and the changes in skin conductance are recorded by EDA in real time.

Before the start of the experiment, the researchers first prepared and calibrated all physiological data acquisition equipment (such as EEG, ECG, and EDA equipment) to ensure that the noise reduction device can generate four different intensities of noise (70 dB(A), 80 dB(A), 90 dB(A), and 40 dB(A)). After the subjects arrived in the laboratory, they filled in the informed consent form and conducted a health assessment to ensure that no related diseases affected the experiment. Then, the subjects were asked to wear physiological equipment, including EEG cap and multi-parameter physiological instrument, meanwhile the researchers checked the equipment to ensure the stability of the signal. Next, according to the order of the Latin square experimental design, the subjects were exposed to four noise levels to avoid the interference of the order effect. Subjects were asked to perform a neurobehavioral ability test using the Neurobehavioral Evaluation System Chinese 3rd Generation (NES-C3) at different levels of noise. It was a computerized task that assessed core neurobehavioral functioning at various noise levels, including the following tests.

(1) Attentional breadth

A countdown was presented at the beginning of the test, and after the countdown was completed, a number of white dots appeared on the screen, which disappeared immediately after presentation, and the subjects were asked to answer the number of white dots presented.

(2) Visual retention

At the beginning of the test, one target picture was presented, which disappeared immediately after presentation, and then four more pictures were displayed, from which they quickly searched for pictures that were consistent with the target picture.

(3) Visual complex reaction time

A target icon appeared randomly on the screen at the start of the test. The subject was asked to press a key as soon as the target icon appeared.

(4) Space perception

Five pictures were presented at the beginning of the test, of which the center picture of the screen was the target picture, and the other four pictures were quickly searched for the picture that was consistent with the target picture.

**Figure 2:**
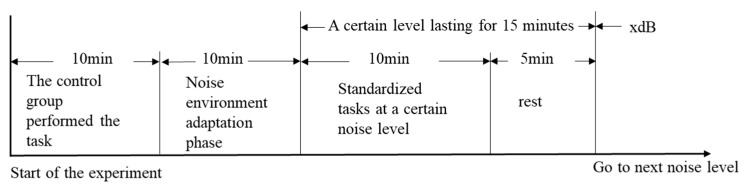
The experimental flow.

The experimental flow is shown in Figure 2.

While the subjects were completing the standardized task, EEG, ECG, and EDA data were collected in real time. After the experiment, the data were supposed to be cleaned and preprocessed. Finally, the effects of different noise sound pressure levels on physiological indicators were evaluated by statistical analysis.

### 2.3. Physiological Signal Processing

**Figure 3:**
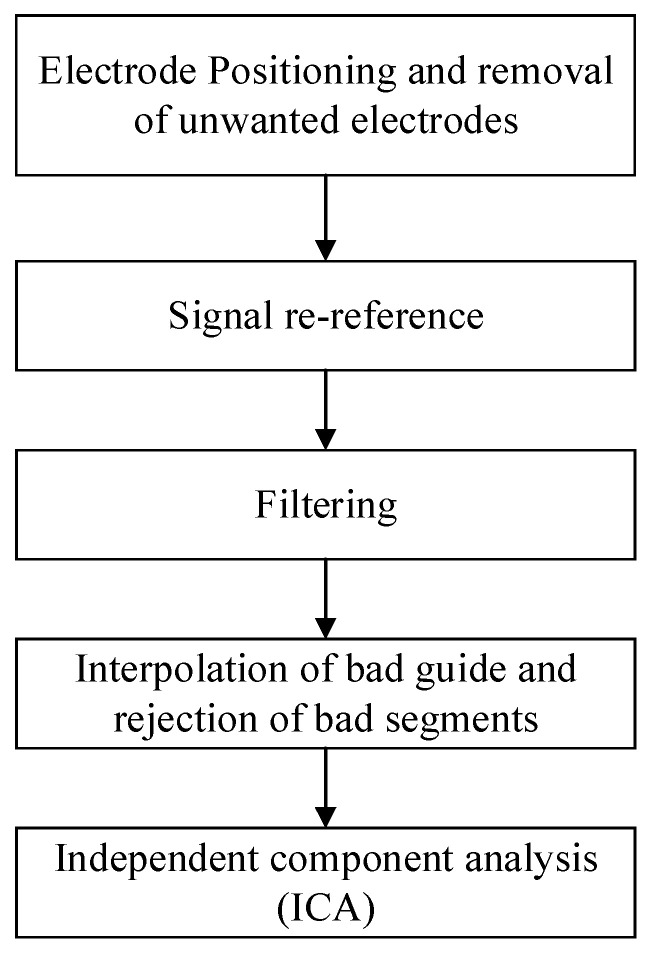
Preprocessing pipeline.

For ECG and EDA data, Starfish software V1 was used to filter and data analyze the raw ECG PICT data; for EEG data, the EEGLAB toolbox of Matlab was used to preprocess the raw EEG data. The pre-processing process of EEG data is shown in Figure 3.

(1) Electrode positioning and removal of unwanted electrodes

After the raw EEG data were imported, the spatial coordinates of the 64-lead standard electrode caps were firstly matched, and 59 valid electrodes were retained for subsequent analysis. During preprocessing, five non-EEG channels (including ECG and ophthalmology channels) were removed: five non-EEG channels, including HEOR, HEOL, VEOL, VEOU, and ECG, were excluded in order to exclude physiological artifacts from interfering with the resolution of the neural signals and to ensure the reliability of the subsequent analyses, and ultimately, 59 valid scalp EEG electrodes were retained for the analyses.

(2) Signal re-reference

In this experiment, the whole-brain average re-reference method was used to subtract the whole-brain average potential from each electrode signal to eliminate the systematic error introduced by the drift of the reference electrode potential.

(3) Filtering

In this experiment, high-pass filtering and low-pass filtering were combined: the cutoff frequency of high-pass filtering was set at 0.5 Hz to remove the baseline fluctuation caused by low-frequency drift and sweat secretion; the cutoff frequency of low-pass filtering was set at 40 Hz to suppress the high-frequency electromyographic noise and power supply frequency interference.

(4) Interpolation of bad guide and rejection of bad segments

For the abnormal signal quality problem during EEG signal acquisition, automated detection algorithms identify and flag “bad segments” of data that are of substandard quality or have obvious anomalies; for the “bad conductor” problem, due to poor electrode contact, an interpolation algorithm based on spatial topology was used.

(5) Independent component analysis (ICA)

For acquired continuous signals, the raw data are decomposed using ICA to eliminate relevant components labeled as artifacts.

## 3. Results

### 3.1. The Effect of Noise on Electroencephalogram (EEG)

**Figure 4:**
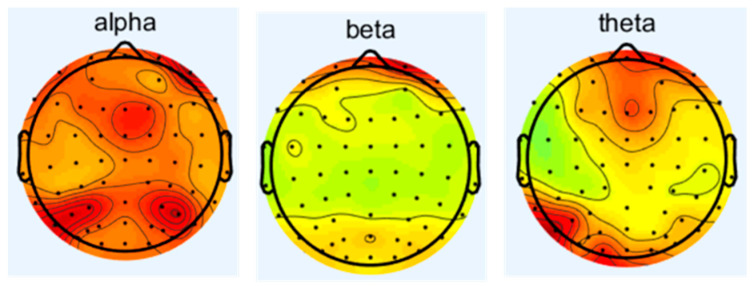
Brain topographic map at 40 dB(A) noise level.

**Figure 5:**
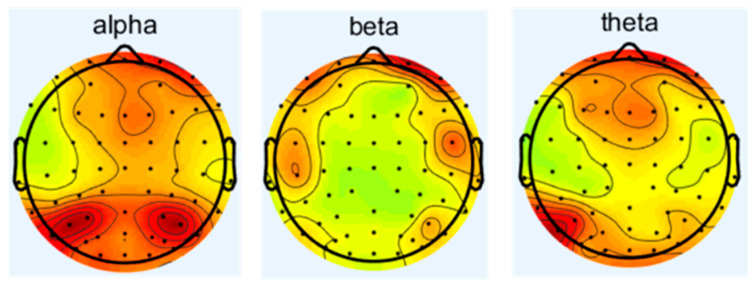
Brain topographic map at 70 dB(A) noise level.

**Figure 6:**
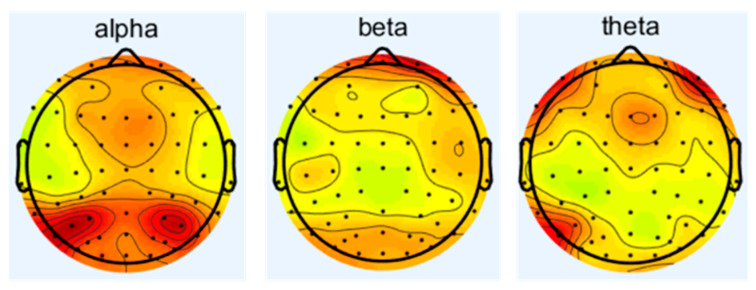
Brain topographic map at 80 dB(A) noise level.

**Figure 7:**
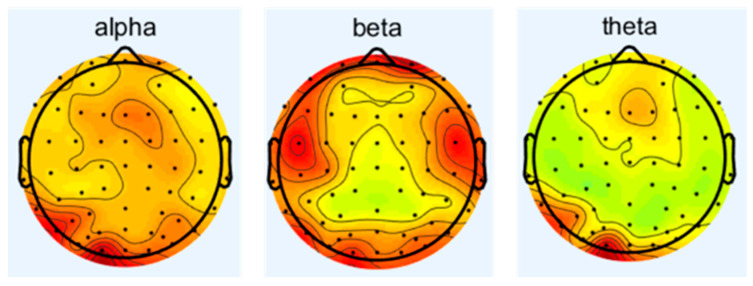
Brain topographic map at 90 dB(A) noise level.

Since the condition of δ wave is in human sleep or anesthesia, this experiment is conducted while the subjects are awake and focused; therefore, this study focuses on alpha, beta, and theta waves to investigate the effects of noise on the central nervous system. The spectrum analysis of EEG is carried out to obtain the difference of energy of different frequency waves under different sound levels of noise. The changes in the frequency waves of 16 subjects under different working conditions are as follows in Figure 4, Figure 5, Figure 6 and Figure 7, which represent the brain topography with noise levels of 60 dB(A), 70 dB(A), 80 dB(A), and 90 dB(A), respectively. It can be seen from the figure that as the noise level increases from 40 dB(A) to 90 dB(A), α-wave suppression is mainly concentrated in the combined area of the occipital lobe and parietal lobe, which is usually related to visual information integration and resting state attention maintenance. The enhancement of the β wave is concentrated in the frontal lobe, suggesting that noise stimulation activated the executive control network and motor preparation system. The β-wave enhancement was concentrated in the frontal lobe, suggesting that the noise stimulus activated the executive control network and the motor preparation system, indicating that the subjects actively mobilized their working memory and decision-making resources to counteract the noise interference in order to maintain the operational efficiency; theta-wave inhibition was dominated by the frontal lobe and the temporal lobe, and the reduced power may reflect that the noise environment suppressed the spontaneous neural activity of the brain in the resting state, which led to the redeployment of more cognitive resources into the processing of the task in response to the external stimulus. The reduced power may reflect that the noise environment inhibits the brain’s spontaneous neural activity in the resting state, allowing more cognitive resources to be reallocated to task processing in response to external stimuli.

**Figure 8:**
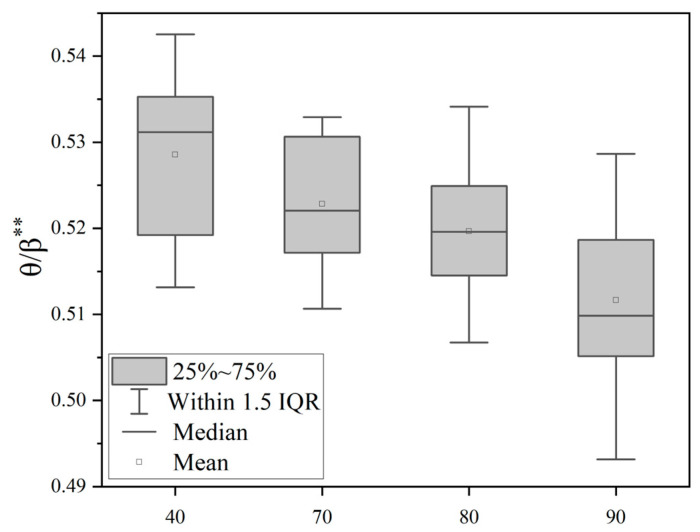
Boxplot of the θ/β ratio across different noise sound pressure levels. Note: ** *p* < 0.01.

**Figure 9:**
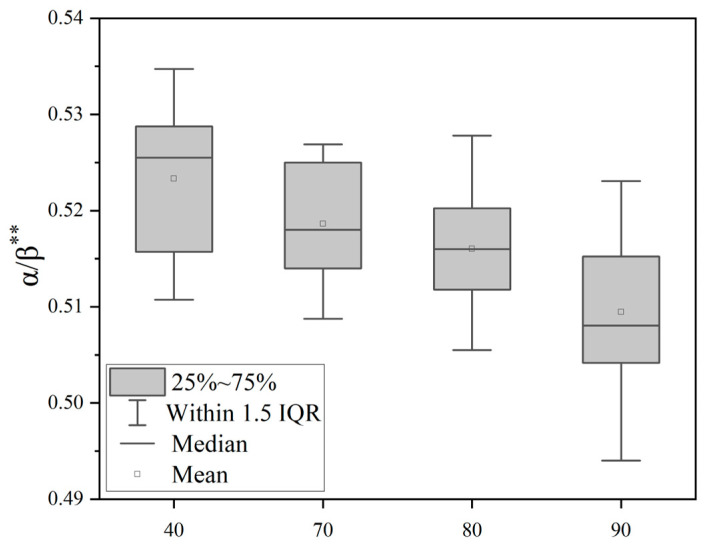
Boxplot of the α/β ratio across different noise sound pressure levels. Note: ** *p* < 0.01.

**Figure 10:**
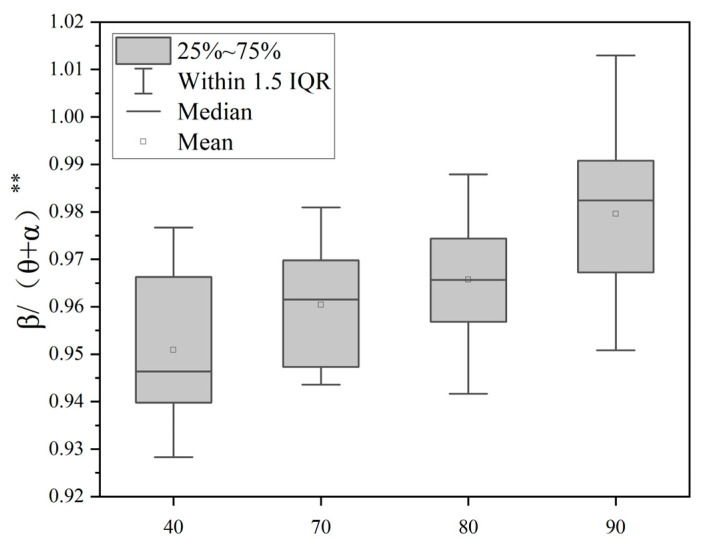
Boxplot of the β/(θ + α) ratio across different noise sound pressure levels. Note: ** *p* < 0.01.

Through the single-factor variance analysis of θ/β, α/β, β/(θ + α) indicators, as shown in Table 1, the difference comparison of the EEG indicators under different noise levels can be obtained. It can be seen that the EEG indicators measured under different noise levels, θ/β, α/β, β/(θ + α), have significant differences and statistical significance. Figure 8 shows that the θ/β ratio is significantly different (*p* < 0.01) between quiet conditions (40 dB(A)) and noisy environments (70–90 dB(A)), and tends to decrease with an increasing noise sound pressure level. Figure 9 shows that the α/β ratio is significantly different between noise groups (*p* < 0.01) and shows a decreasing trend with an increasing noise sound pressure level. Figure 10 is a boxplot of the β/(θ + α) ratio across different noise sound pressure levels which shows that the β/(θ + α) ratios are significantly different between noise groups (*p* < 0.01) and shows an increasing trend with an increasing noise sound pressure level.

### 3.2. The Effect of Noise on Electrocardiogram (ECG)

During the data analysis of this study, the normality test revealed that the distribution of the data in each group deviated significantly from the normal distribution and the residual analysis showed that the assumption of the variance chi-square was not valid, so the data were analyzed using a nonparametric statistical framework.

The overall difference between the four noise levels was tested using the Freedman’s test, and the results in Table 2 show statistically significant differences in the ECG metrics.

**Figure 11:**
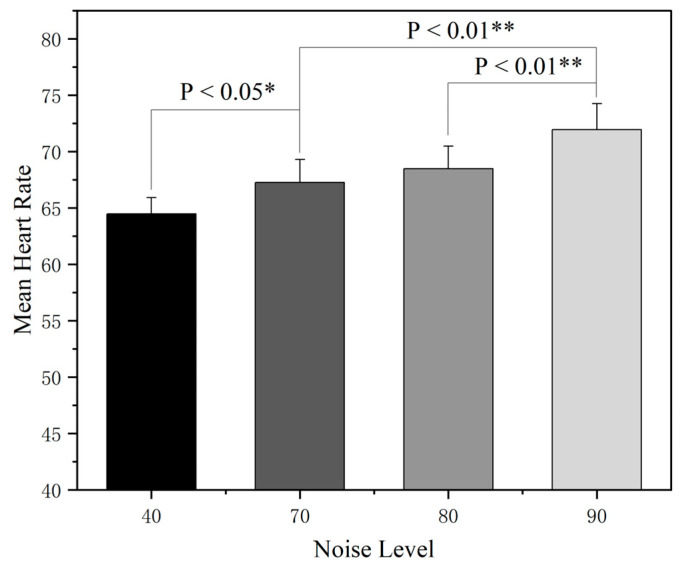
Heart rate rank-sum test across different noise sound pressure levels. Note: * *p* < 0.05, ** *p* < 0.01.

The effect of different noise levels on the participants’ heart rate levels was further assessed by a nonparametric rank-sum test system. The analysis of the heart rate data is shown in Figure 11. The heart rate rank-sum test across different noise sound pressure levels revealed that there was a statistically significant difference in heart rate values between the quiet condition and the noisy environment, and the average heart rate of the participants showed a stepwise incremental trend as the noise level was gradually increased from 40 dB(A) to 90 dB(A), with the differences between the 70 dB(A) and 90 dB(A) groups and the 80 dB(A) and 90 dB(A) groups reaching the level of high significance (*p* < 0.01), indicating that the stimulation effect on the autonomic nervous system was further expanded after the noise intensity broke through the specific threshold.

In the previous heart rate analysis, it was found that noise exposure elevated the heart rate of subjects, but the change in the absolute value of the heart rate was not yet sufficient to fully reveal the effect of noise on the autonomic nervous system. To refine the effects of noise on autonomic function, the present study introduced a time-domain indicator of heart rate variability: the Standard Deviation of Normal-to-Normal intervals (SDNN) and the Percentage of adjacent NN intervals differing by >50 ms (PNN50) were analyzed in depth.

**Figure 12:**
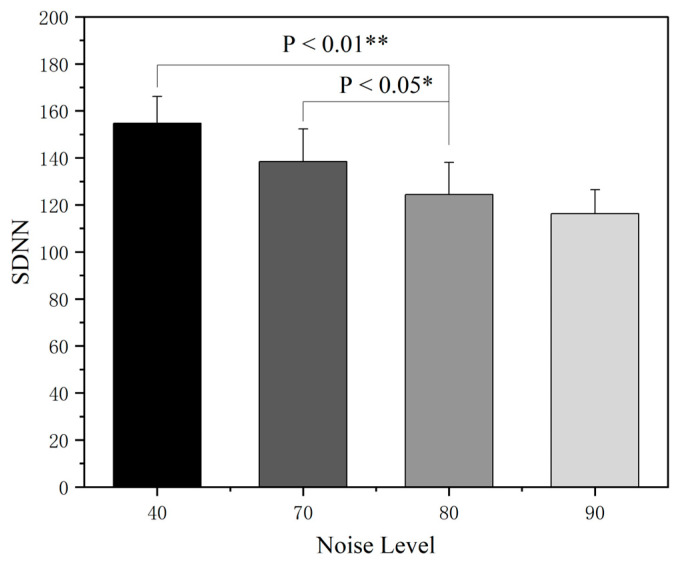
SDNN rank-sum test across different noise sound pressure levels. Note: * *p* < 0.05, ** *p* < 0.01.

Figure 12 shows an in-depth analysis of the effects of different noise intensities on the SDNN, a time-domain indicator of heart rate variability, by means of a nonparametric rank-sum test. The data analysis showed that when the noise intensity reached 80 dB(A), the SDNN values in quiet conditions and noise-exposed environments showed highly significant differences, and the SDNN showed a systematic decreasing trend with an increasing noise intensity, suggesting that noise exposure can significantly weaken the overall regulatory ability of the autonomic nervous system. It is worth noting that the difference in the SDNN between the 70 dB(A) and 80 dB(A) and above noise groups was statistically significant, whereas the decrease in the SDNN between the 80 dB(A) and 90 dB(A) groups did not reach the threshold of significance, which is a nonlinear feature suggesting that the autonomic nervous system may enter a compensatory state after 80 dB(A).

**Figure 13:**
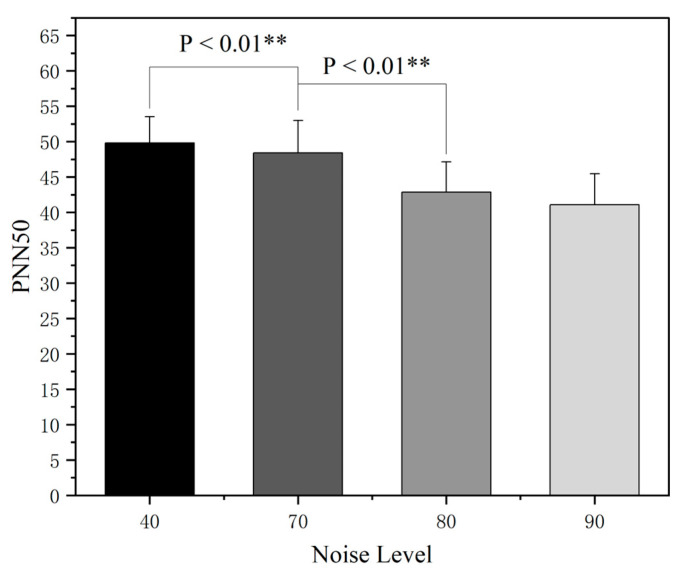
PNN50 rank-sum test across different noise sound pressure levels. Note: ** *p* < 0.01.

Figure 13 shows the nonparametric rank-sum test analysis of different noise intensities on PNN50, a time domain indicator of heart rate variability (HRV), which systematically explored the effects of different noise levels on PNN50, an indicator of heart rate variability. The data analysis showed that there was a significant difference between the PNN50 values in quiet conditions and noise-exposed environments, and the indicator showed a continuous decay trend as the noise intensity increased from 40 dB(A) to 90 dB(A) in a gradient, confirming that high-intensity noise could suppress parasympathetic nerve activity. Specifically, the difference in PNN50 between the 70 dB(A) and 80 dB(A) noise groups was statistically significant, whereas the decrease in PNN50 between the 80 dB(A) and 90 dB(A) groups was not significant, suggesting that the process of the inhibition of parasympathetic activity may enter into a compensatory saturation state after 80 dB(A).

### 3.3. Effect of Noise on Electrodermal Activity (EDA)

Studies have shown that skin electrical activity increases when emotional stress and anxiety are enhanced, indicating that the tension of the autonomic nervous system increases [24]. In this study, skin conductance (SC) is selected as the measurement indicator of EDA. Table 3 can be obtained by completing a one-way analysis of variance on the EDA data. It can be obtained from the table that there are significant differences in the SC level under different noise conditions, which is statistically significant.

**Figure 14:**
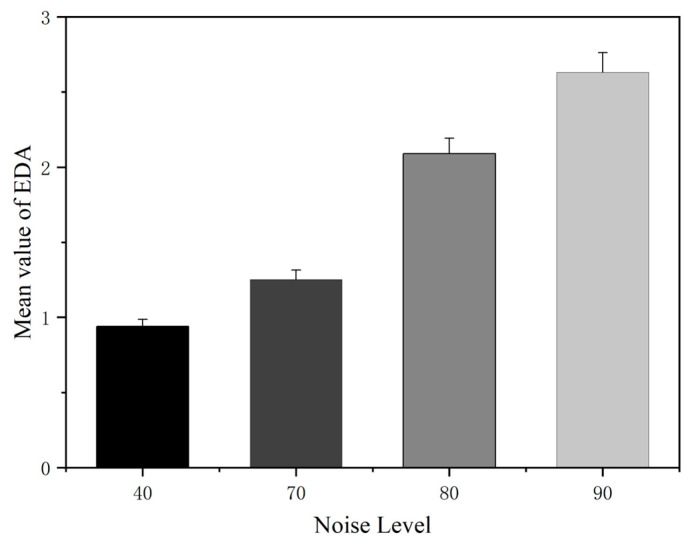
The error bar chart of EDA indicator under different noise levels.

It can be seen from Figure 14 that with the increase in the noise level, the skin electrical indicators of the subjects showed an upward trend. This is because noise, as a stress source, directly activates the sympathetic nervous system, promotes the secretion of sweat glands, and leads to the increase in the electrolyte concentration on the skin surface, which is manifested as the increase in skin conductivity.

### 3.4. Correlation Analysis Between Physiological Indicators

**Figure 15:**
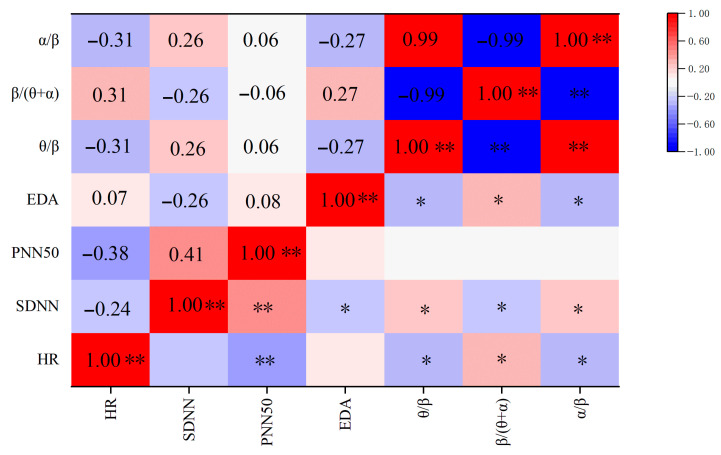
The correlation heat map between physiological indicators. Note: * *p* < 0.05, ** *p* < 0.01.

As shown in Figure 15, rows and columns are physiological indicators of flying cadets. The Pearson correlation coefficient between each factor is calculated by SPSS IBM SPSS Statistics 27.0.1 (r value range: −1 to 1). The red color of the correlation heat map shows a positive correlation (r > 0) and the blue color shows a negative correlation (r < 0). The color depth is proportional to the value of |r| (light color: |r| < 0.3; deep color: |r| > 0.6).

Through the heat map, it can be obtained that the heart rate is negatively correlated with SDNN (r = −0.244) and PNN50 (r = −0.383 **). This is because with the increase in the noise level, sympathetic nerve activation (heart rate acceleration), and the inhibition of parasympathetic nerve activity (HRV reduction), the SDNN and PNN50 decrease synchronously; there is a strong positive correlation between the SDNN and PNN50 (r = 0.410 **), both of which belong to the time domain indicator of HRV. The decrease in the SDNN represents the enhancement of cardiac sympathetic nerve activity, and PNN50 represents the imbalance of autonomic nerve function. Noise may lead to the simultaneous decrease in the two by inhibiting the vagus nerve.

The negative correlation between the heart rate and θ/β indicator and α/β indicator (r = −0.31 *) indicates that noise inhibits θ wave and α wave activity and enhances β wave activity (θ/β, α/β ratio decreased) by activating the sympathetic nerve (heart rate electrical indicator changes), reflecting the dynamic redistribution of the autonomic nervous system and brain cognitive resources under stress.

The negative correlation between the EDA and SDNN (r = −0.26 *) indicated that the increase in EDA (sympathetic nerve excitation) is correlated with the decrease in the SDNN (autonomic nerve regulation weakening), which is consistent with the characteristics of a noise-induced stress response. The reason is that noise stimulation activated the sympathetic nerve, resulting in increased skin electrical conductivity, an inhibited parasympathetic nerve, and reduced HRV.

The negative correlation between skin conductance and the θ/β indicator and α/β indicator (r = −0.27 **) indicates that the increase in skin conductance (enhanced stress) is accompanied by the decrease in the θ/β indicator value and α/β indicator value, reflecting that noise, as a pressure source, will activate the sympathetic nervous system and interfere with the EEG rhythm, reflecting the coupling relationship between autonomic nervous activity and brain cognitive resource regulation under stress.

## 4. Discussion

The study by Heow Pueh Lee et al. showed that the cabin of an aircraft is exposed to more than 70 dB(A) during takeoff and landing, with some exceeding 80 dB(A), and the noise level remains above 70 dB(A) during cruising [2]. Based on this research result, this paper sets the independent variable, the noise pressure level, at four levels: 40 dB(A), 70 dB(A), 80 dB(A), and 90 dB(A), with 40 dB(A) as the control group. This setting condition includes all the noise environments that the aircraft may experience, aiming to comprehensively study the effects of different noise pressure levels on pilots’ physiological indicators and provide a theoretical basis for noise airworthiness standards.

As for EEG indicators, although different noise levels have been analyzed to show significant effects on the power of the fundamental frequency bands such as θ, α, and β, it is often difficult for these single-band metrics to comprehensively reflect the complex regulatory mechanisms of brain functional states. Therefore, this study further introduced ratio-type metrics such as θ/β, α/β, and β/(θ + α) as sensitive characterizations of a comprehensive cognitive load, physiological arousal, or an attentional state commonly used in the EEG frequency domain.

As for the ECG indicators, the average heart rate increased, indicating that the subject’s body is in a state of increased tension, stress, or excitement, rather than a state of relaxation and rest [25]; PNN50 (the proportion of heart beats with an adjacent normal RR interval difference > 50 ms) reflects the ability of the vagus nerve to regulate the heart rate. Its decrease indicates that the activity of the vagus nerve is weakened and the sympathetic nerve is relatively hyperactive, which is common in chronic stress or anxiety. SDNN is the standard deviation of RR interval. The decrease in the SDNN indicates that the overall regulation ability of the heart is weakened, which is closely related to the imbalance of the sympathetic–vagus nerve [26]. When the PNN50 and SDNN decrease at the same time, it suggests that the cardiac autonomic nerve function is seriously impaired. Through this study, it was found that with the change in the noise sound pressure level, the physiological indicators of the pilot trainees produced significant changes, i.e., the heart rate tended to increase with the increase in the noise level. Also, the step PNN50 and SDNN tended to decrease, which indicated that the autonomic function of the subjects was affected by the noise, and the activity of the vagus nerve was weakened in a state of discomfort and anxiety.

Previous studies have shown that noise can have an impact on the auditory and cardiovascular systems of organisms, thereby affecting their physiological indicators. Different from other organisms, noise can induce psychological anxiety and other emotions in humans, making the mechanism of noise’s impact on physiological indicators more complex [27,28]. Through this study, it is found that as the sound pressure level of noise changes, the physiological indicators of pilot trainees undergo significant changes. The reason is that noise first affects the auditory system and then transmits to the central nervous system (such as the brain) through the auditory nerve, leading to changes in brain wave patterns, especially in the activity of alpha and beta waves, manifested as a decrease in attention and cognitive ability, followed by the activation of the sympathetic nervous system. This has a direct impact on electrocardiogram (ECG) signals and electrodermal activity (EDA) signals, manifested as changes in physiological indicators, such as the heart rate, PNN50, and SDNN, and skin conductance (SC), thereby potentially affecting human health. The research results have demonstrated the multidimensional damage of cockpit noise to pilots’ physiological systems, providing a scientific basis for establishing cockpit noise limits. In addition, in this study of correlation, weak correlations were found between physiological indicators of different classes, which may indicate that multiple physiological metrics can be jointly modeled or jointly analyzed to construct the pilot’s stress response to noise from multiple dimensions.

Given that this study used headphones, amplifiers, and sound cards to reconstruct the sound field, noise exposure was largely limited to the auditory path. While this approach facilitated the control of acoustic parameters, it also meant that the non-auditory components were not covered. Future studies should consider introducing body vibration or low-frequency sound fields to more fully assess the effects of noise on physiological indicators. It is worth noting that although the experimental subjects in the present study were pilot trainees undergoing professional training, their physiological responses may differ from those of professional pilots with many years of flight experience, and those with more flight experience may show greater neurological adaptability and psychological tolerance in the face of environmental stresses such as cockpit noise. Therefore, some caution should be exercised when directly extrapolating the current results to the entire pilot population, especially the highly experienced group. Future studies may consider introducing samples of pilots from different experience levels to further compare their physiological responses to cockpit noise, in order to enhance the representativeness and explanatory power of the results.

## 5. Conclusions

Through the above research, combined with the results of the data analysis, the following conclusions can be drawn.

(1) The increase in the cockpit noise level will activate the sympathetic nervous system to induce a stress response, promote the secretion of related stress hormones, directly lead to abnormal cardiovascular indicators such as an accelerated heart rate, and increase sweat gland secretion, thus indirectly affecting the skin conductance level.
(2) Cockpit noise acts on the central nervous system through the auditory pathway, interferes with the balance between the excitation and inhibition of the cerebral cortex, and leads to changes in the power spectral density of the main frequency of EEG (such as α wave, β wave), which is manifested as the inhibition of the α wave (8–13 Hz) and enhancement of the β wave (14–30 Hz).
(3) Based on the Pearson correlation analysis, several physiological indicators of pilot trainees in the noise environment exhibited significant correlations. While strong correlations were observed within certain individual indicators, the correlation coefficients (|r|) between different types of indicators generally ranged from 0 to 0.5, indicating low-to-moderate levels of association.

## Figures and Tables

**Table 1:** Difference comparison of EEG indicators under different noise levels.

EEG	N	F	P
θ/β	16	10.624	0.000 **
α/β	16	10.704	0.000 **
β/(θ + α)	16	10.684	0.000 **

Note: ** *p* < 0.01.

**Table 2:** Comparison of ECG indicators under different noise levels.

Null Hypothesis	Test	Significance	Decision
The distributions of heart rate under 40 dB(A), 70 dB(A), 80 dB(A), and 90 dB(A) are identical.	Related-sample Friedman’s two-way analysis of variance by ranks	*p* = 0.000 **	Reject the null hypothesis.

Note: ** *p* < 0.01.

**Table 3:** The difference analysis of EDA indicator SC under different noise levels.

Noise Level	N	$\bar{x}\;$± s	F	*p*
40	16	0.94 ± 1.46	3.245	0.029 *
70	16	1.25 ± 1.48		
80	16	2.09 ± 1.78		
90	16	2.63 ± 1.89		

Note: * *p* < 0.05.

## Data Availability

The data presented in this study are available on request from the corresponding author as the indicators used in this experiment are physiological indicators of pilot trainees, which are private data of the participants, so they cannot be provided.
